# Improved body composition decreases the fat content in non-alcoholic fatty liver disease, a meta-analysis and systematic review of longitudinal studies

**DOI:** 10.3389/fmed.2023.1114836

**Published:** 2023-05-04

**Authors:** Dóra Mátis, Péter Hegyi, Brigitta Teutsch, Tamás Tornai, Bálint Erőss, Gabriella Pár, Szilárd Váncsa

**Affiliations:** ^1^Centre for Translational Medicine, Semmelweis University, Budapest, Hungary; ^2^Institute for Translational Medicine, Medical School, University of Pécs, Pécs, Hungary; ^3^Institute of Pancreatic Diseases, Semmelweis University, Budapest, Hungary; ^4^Division of Gastroenterology, First Department of Medicine, Medical School, University of Pécs, Pécs, Hungary

**Keywords:** non-alcoholic fatty liver disease, metabolic syndrome, weight loss, muscle mass, fat mass

## Abstract

**Background:**

Based on cross-sectional studies, there is a link between body composition parameters and steatosis in non-alcoholic fatty liver disease (NAFLD). However, whether long-term changes in different body composition parameters will result in NAFLD resolution is unclear. Therefore, we aimed to summarize the literature on longitudinal studies evaluating the association between NAFLD resolution and body composition change.

**Methods:**

Based on the recommendations of the Cochrane Handbook, we performed a systematic search on September 26th, 2021, in three databases: Embase, MEDLINE (via PubMed), and Cochrane Central Register of Controlled Trials (CENTRAL). Eligible studies reported on patients with NAFLD (liver fat >5%) and examined the correlation between body composition improvement and decrease in steatosis. We did not have pre-defined body composition or steatosis measurement criteria. Next, we calculated pooled correlation coefficient (*r*) with a 95% confidence interval (CI). Furthermore, we narratively summarized articles with other statistical methods.

**Results:**

We included 15 studies in our narrative review and five in our quantitative synthesis. Based on two studies with 85 patients, we found a pooled correlation coefficient of *r* = 0.49 (CI: 0.22–0.69, Spearman's correlation) between the change of visceral adipose tissue and liver steatosis. Similarly, based on three studies with 175 patients, the correlation was *r* = 0.33 (CI: 0.19–0.46, Pearson's correlation). On the other hand, based on two studies with 163 patients, the correlation between subcutaneous adipose tissue change and liver steatosis change was *r* = 0.42 (CI: 0.29–0.54, Pearson's correlation). Furthermore, based on the studies in the narrative synthesis, body composition improvement was associated with steatosis resolution.

**Conclusions:**

Based on the included studies, body composition improvement may be associated with a decrease in liver fat content in NAFLD.

**Systematic review registration:**

Identifier: CRD42021278584.

## 1. Introduction

Non-alcoholic fatty liver disease (NAFLD) has emerged as one of the most common causes of chronic liver disease worldwide and represents a global public health problem. It already affects 25% of the global population, and the prevalence is predicted to grow rapidly (1).

Despite its increasing prevalence and clinical importance, NAFLD's pathogenesis and optimal management are poorly understood. Evidence-based practice guidelines reserve pharmacologic treatment for patients with advanced fibrosis or active necro-inflammation at high risk of progression (2). Although several pharmacological agents have been evaluated (e.g., pioglitazone, vitamin E, liraglutide, semaglutide), no drugs have been included in the guidelines yet (3, 4).

Current guidelines agree that lifestyle changes, including dietary interventions and physical exercise, remain the cornerstone in NAFLD management, and these interventions should be implemented as a first-line measure in all patients (2). Research shows a dose-dependent relationship between weight loss and non-alcoholic steatohepatitis (NASH) resolution, meaning that a modest (5%−7%) weight loss can already promote the decrease of steatosis. However, a considerable weight loss (>7%−10%) is necessary to maximize the benefit (5, 6). Multiple exercise-based interventional studies proposed a new perspective by demonstrating that the decrease of intrahepatic lipid content can be achieved without substantial weight loss (7). A study by Kabir et al. (8) highlighted that in patients with NAFLD, the type of fat and its regional distribution seem to be more important than the absolute amount.

Based on previous studies, the severity of liver steatosis is negatively affected by increased visceral adipose tissue (VAT), subcutaneous adipose tissue (SAT), and decreased skeletal or appendicular muscle mass (9). However, most studies on this topic were cross-sectional and could not demonstrate a causal relationship between these parameters and NAFLD resolution (9). Recently, body composition has become a central issue in many longitudinal trials about NAFLD, focusing on VAT, SAT, total fat mass, or lean muscle mass improvement and change in liver steatosis (10, 11). These studies reported on the relationship between the improvement of body composition parameters and regression of liver steatosis.

In the present systematic review and meta-analysis, we aimed to explore the results of the available longitudinal studies of how the alteration of body composition parameters such as VAT, SAT, and muscle mass correlates with the change in liver steatosis and NAFLD regression.

## 2. Methods

We report our systematic review and meta-analysis based on the PRISMA 2020 recommendations (12) (see Supplementary Table 1) while following the Cochrane Handbook (13). The study protocol was registered on PROSPERO (registration number CRD42021278584). However, we decided not to exclude papers based on the statistical methods used. Therefore, we included each study corresponding with our research question.

### 2.1. Information sources and search strategy

Our systematic search was conducted on September 26^th^, 2021, in three databases: Embase, MEDLINE (via PubMed), and Cochrane Central Register of Controlled Trials (CENTRAL). We used the following search key in all databases: (“Fatty liver disease” OR steatohepatitis OR steatosis OR NAFLD OR NASH OR MAFLD) AND ((fat OR obes^*^ OR adipos^*^) AND (visceral OR “intra abdominal” OR abdominal OR central) OR “fat mass” OR “skeletal mass” OR SMI OR “body composition” OR “muscle mass” OR “fat free mass” OR “body fat”). No filters were applied during the search. Finally, we searched the reference list of eligible studies.

### 2.2. Eligibility criteria

We formulated our research question using the PFO format (patients, prognostic factor, and outcome). We included longitudinal studies which examined the association between the change of various body composition parameters and the change of hepatic steatosis in adult participants (P) with NAFLD. Steatosis was defined as ≥5% intrahepatic lipid content at baseline (2). This widely accepted diagnostic approach served as a comprehensive definition for baseline hepatic status since various diagnostic methods, and steatosis definitions appear in the studies. The F from the PFO format represents the change in body composition [e.g., body fat (BF), fat-free mass (FFM), skeletal muscle mass (SMM), visceral fat (VF), VAT, or SAT]. In eligible studies, the main objective was to achieve body composition change. Therefore, we did not emphasize the means of accomplishing it.

The primary outcome was the change in liver fat content from baseline to the end of follow-up (O), described as, e.g., intrahepatic liver fat, Fibroscan Controlled Attenuation Parameter (CAP), NASH score, Hepatic Steatosis Change, and NAFLD resolution. In addition, eligible studies either reported the correlation between body composition change and liver fat content change, the mean of body composition parameters, or the resolution of NAFLD as a dichotomous outcome.

We did not use a pre-defined body composition measurement (e.g., bioelectrical impedance analysis—BIA, Dual-energy X-ray absorptiometry—DXA, computed tomography—CT, or air displacement plethysmography—ADP) and liver fat content measurement (e.g., proton magnetic resonance spectroscopy—H-MRS, magnetic resonance imaging—MRI, or ultrasound—US) methods.

Lastly, the following exclusion criteria were used: (1) studies reported on patients undergoing bariatric surgery, (2) case reports or series and other descriptive studies, (3) studies with a mean follow-up period of fewer than 2 months, (4) studies containing data only on the conventional anthropometric measure (BMI, BMI *z*-score, BMI percentile, waist-to-height ratio), and (5) cross-sectional studies.

### 2.3. Selection process

After duplicate removal, the selection was performed by two independent review authors (DM and SV) by title, abstract, and full-text based on pre-discussed aspects. We used Endnote v9.0 (Clarivate Analytics, Philadelphia, PA, USA) reference manager software for the selection. Disagreements were resolved by consensus, and if consensus was not reached, a third independent review author (BT) was involved in deciding.

### 2.4. Data collection process and data items

Two authors (DM and BT) independently collected data from the eligible articles. In the case of disagreement, the decision was based on consensus, or if it was not reached by involving a third author (SV).

The following data were extracted: first author, the year of publication, study population, study period, study site (country), study design, demographic data of the patients, total follow-up time, type of intervention (if applicable), method of measurement of hepatic steatosis and body composition parameters, body composition and outcome parameters (as defined in the article), correlation coefficients between the change of steatosis and different body composition parameters, and information for assessing the risk of bias in the study.

### 2.5. Study risk of bias assessment

Two authors performed the risk of bias assessment independently with the help of the Quality in Prognostic Studies (QUIPS) tool (14). A consensus was reached in the case of disagreements. The specific methodological details are described in Supplementary Appendix 1. The “study attrition” domain was omitted in the case of retrospective studies. The web-based version of the Risk Of Bias VISualization (ROBVIS) tool was used to visualize the results (15).

### 2.6. Synthesis methods

Due to the heterogeneity of the statistical methods used in the included studies examining the relationship between the change of different body composition parameters and the change in liver fat content, we decided to pool only the correlation coefficients. Other results were included in the systematic review part.

We decided to pool data from a minimum of two studies. However, we interpreted the results with limitations. The statistical analysis of the data was conducted using the R programming language [(16), Vienna, Austria, R version 4.1.0]. Using each study's extracted correlation coefficient (*r*), we calculated pooled correlation coefficients with 95% confidence intervals (CIs). Correlation coefficient values were converted by Fisher's *r*-to-*z* transformation to obtain approximately normally distributed *z* values to calculate 95% CIs (17). The random-effects model was used for the pooled analysis in this study. Correlations were classified as weak (*r* = 0–0.30), moderate (*r* = 0.30–0.70), and strong (*r* = 0.70–1.0) (18). A *p*-value of < 0.05 was considered to be statistically significant. We tested heterogeneity with *I*^2^ and χ^2^ tests, and a *p*-value < 0.1 was considered significant heterogeneity.

We grouped the results based on the body composition parameter and the correlation analysis method (Pearson, Spearman, or not defined).

Because of the low number of eligible studies (< 10), we could not assess publication bias.

## 3. Results

### 3.1. Search and selection

**Figure 1:**
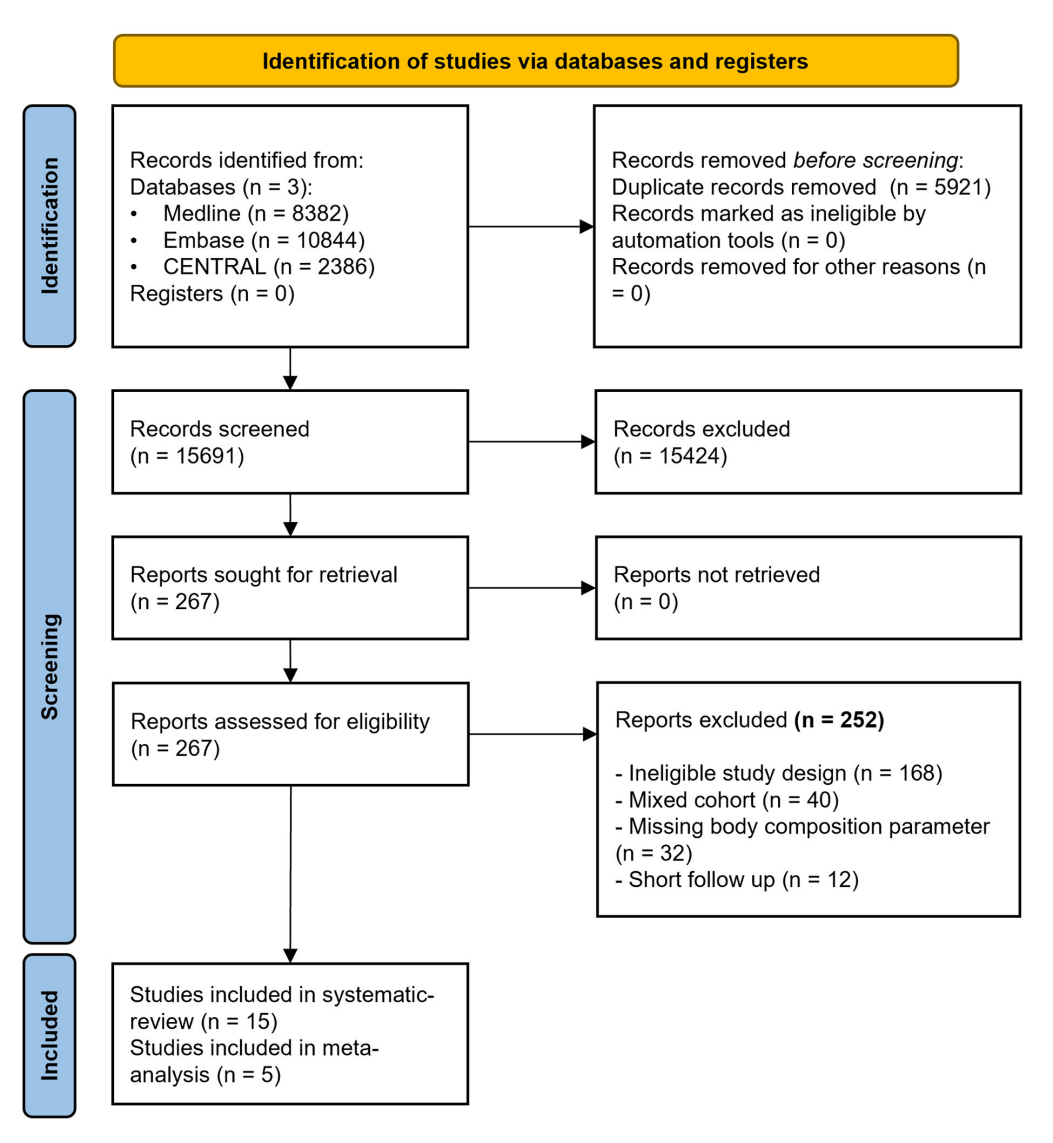
PRISMA 2020 flowchart.

Altogether 21,612 studies were identified by our search, from which 15 full-text articles were included in our synthesis and meta-analytical calculations (10, 11, 19–31). Details of the selection process are presented in the PRISMA flowchart (Figure 1).

### 3.2. Basic characteristics of included studies

The baseline characteristics of the eligible studies are detailed in Table 1. In Supplementary Table 2, we summarized the eligibility criteria extracted from each included article. Regarding geographical localization, four studies originated from Japan (10, 26, 30, 31), three from Korea (11, 25), two from the United Kingdom (20, 21) and the USA (22, 29), and one from Thailand (19), Croatia (24), Australia (23), and Belgium (28). Out of the 15 studies, 12 were prospective cohort studies. The follow-up period for the liver steatosis change varied between 2 and 27 months. Liver steatosis was diagnosed using H-MRS, MRI, US, elastography by Fibroscan CAP, and liver biopsy. Regarding body composition assessment, the most frequently used method was MRI (in five studies), but also other methods such as BIA, DXA, CT, ADP, and InBody 720 were used. Further details of the interventions performed to achieve NAFLD regression are detailed in Supplementary Table 3.

**Table 1:** Basic characteristics of the included studies in the systematic review and meta-analysis.

**References**	**Study site**	**Number of patients (female %)**	**Age (year)^†^**	**Baseline BMI (kg/m^2^)^†^**	**Waist circumference (cm)**	**Body composition measurement**	**Body composition parameter(s)**	**Steatosis assessment**	**Follow-up (months)^†^**	**Outcome measure- ment**
Charatcharoenwitthaya et al. (19)^‡^	Thailand	35 (77)	37 (±2)	26.8 (±0.7)	86.6 (±1.6)	Impedance	FM, SM, VAT rating	Elastography	3	Hepatic fat content
Cuthbertson et al. (20)^‡^	United Kingdom	50 (22)	51 (46–59)	30.7 (29.7–30.6)	106 (101–112)	MR	VAT, SAT	H-MRS	4	IHCL
Houghton et al. (21)^‡^	United Kingdom	12 (ND)	54 (±12)	33 (±7)	ND	MR	VAT	H-MRS, biopsy	3	HTGC
Huang et al. (22)^‡^	USA	15 (47)	48.8 (±12)	34 (±7)	106.6 (±13)	CT	VAT, BF	Biopsy	12	NASH score
Kendel Jovanović et al. (24)^‡^	Croatia	42 (93)	43.6 (±5.8)	35.4 (±4.3)	108.4 (±8.4)	Impedance	VAT, fat tissue %	FL index, NAFLD-FLS	6	HS change
Keating et al. (23)^‡^	Australia	48 (65)	44 (±3)	33.4 (±1.3)	93.7 (±1.5)	MR	VAT, SAT	H-MRS	2	IHL
Kim et al. (25)^‡^	Korea	314 (19)	52.9 (±9)	25.71 (± 2.37)	90.22 (± 6.67)	CT	VAT, SAT	US	55.7	NAFLD resolution
Kim et al. (11)^‡^	Korea	2631 (24)	51.3 (±8.1)	27.1 (±2.3)	94.5 (±7.0)	InBody 720	SM index	HS index	56.1 ± 12.4	NAFLD resolution
Koda et al. (26)^‡^	Japan	28 (71)	53 (±12)	26.4 (±3.3)	ND	Impedance	VAT, SAT	US	27 ± 26	HS score, ALT
Lee et al. (27)	Korea	115 (12)	31 (±7.5)	25.4 (±3.5)	ND	CT	VAT, SAT, SM	Biopsy	3	HS %
Nachit et al. (28)^‡^	Belgium	39 (69)	42.8 (±5.6)	39.8 (±5.5)	ND	CT, impedance	Skeletal muscle fat index	Biopsy	14	NASH improvement
Osaka et al. (10)	Japan	117 (50)	63.5 (±12.2)	25.4 (±4.4)	ND	Inbody 720	Fat-to-muscle ratio	Elastography	12	LSM and CAP
Rachakonda et al. (29)^‡^	USA	52 (79)	47.6 (41.1–52)	45.6 (43.4–48)	127.4 (123.4–131.3)	CT, DXA, plethysmography	FM, FFM, midthigh muscle area, VAT, SAT	CT	6	NAFLD resolution
Shida et al. (30)	Japan	92 (61)	55.5 (±14.3)	27.9 (±5.1)	ND	InBody 720	SV ratio (skeletal MM to visceral fat are)	US, Fibroscan	49.2	AST, ALT, LSM, and CAP
Takahashi et al. (31)^‡^	Japan	28 (71)	56.7 (±12)	28.3 (±3.2)	ND	Impedance	Muscle/body weight %	US	24	ALT

† Mean or median with standard deviation or range.

‡ Prospective study.

ALT, alanine aminotransferase; BF, body fat; BMI, body mass index; CAP, controlled attenuation parameter; DXA, dual-energy X-ray absorptiometry; FFM, fat-free mass; FL, fatty liver; FM, fat mass; H-MRS, Proton Magnetic Resonance Spectroscopy; HS, hepatic steatosis; HTGC, hepatic triglyceride content; IHCL, intra-hepatocellular lipid; IHL, intra-hepatic lipid; LSM, liver stiffness measurement; MR, magnetic resonance; MR-PDFF, MR-Proton density fat fraction; ND, not defined; SAT, subcutaneous adipose tissue; SM, skeletal muscle; SMI, skeletal muscle index; US, ultrasound; USA, United States of America; VAT, visceral adipose tissue.

### 3.3. Visceral adipose tissue area decrease moderately correlates with steatosis improvement

**Figure 2:**
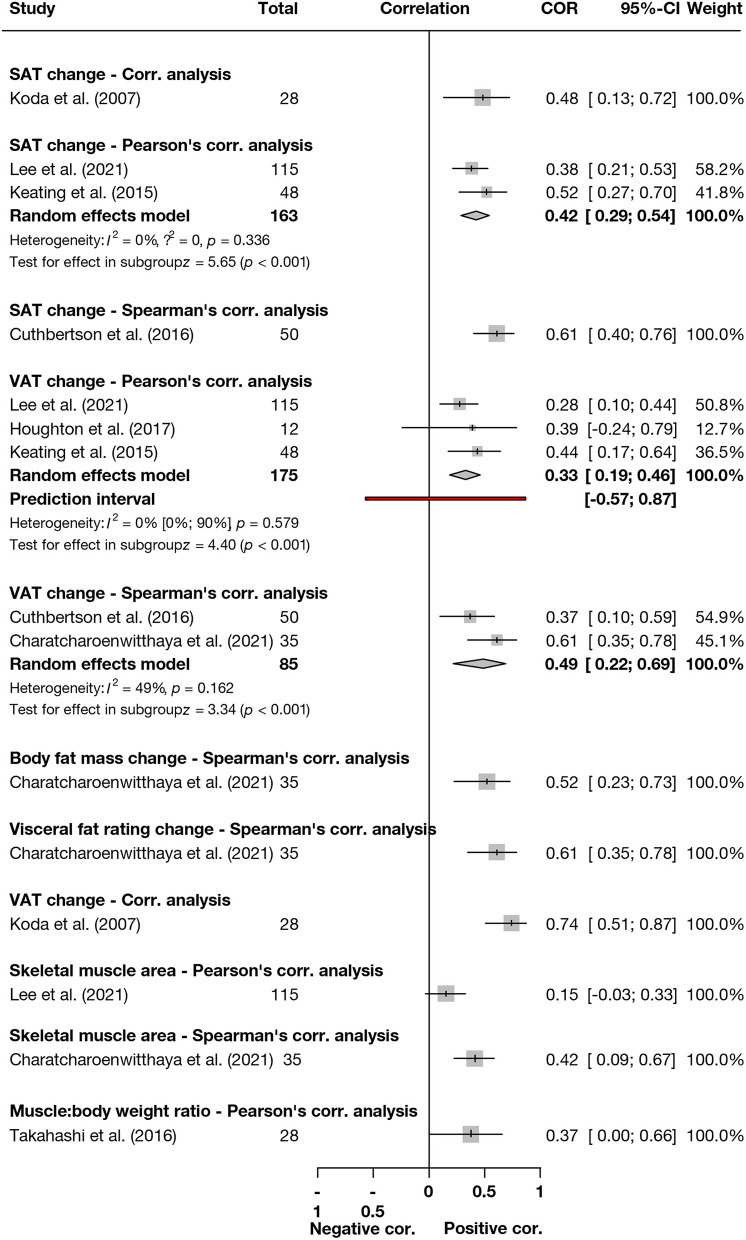
Summary forrest plot presenting different correlation analysis results between body composition parameter changes and liver fat content decrease. SAT, subcutaneous adipose tissue; VAT, visceral adipose tissue.

The results of correlation analyses between liver steatosis and body composition parameters are summarized in Figure 2. Due to the low number of studies included, we interpreted the results with limitations. Based on two studies (19, 20) with 85 patients, we found a pooled correlation coefficient of *r* = 0.49 (CI: 0.22–0.69, Spearman's correlation) between VAT change and liver steatosis. Similarly, based on three (21, 23, 27) studies with 175 patients, the correlation was *r* = 0.33 (0.19–0.46, Pearson's correlation, Figure 2).

Huang et al. (22) recruited patients with NASH for a 1-year intense nutritional counseling, while their primary endpoint was the histologic improvement of NASH, defined as a ≥2-point reduction in the total NASH score (0–17). Patients with an improved score had a mean change in VAT volume of −36.18 cm^3^, while those without improvement had −12.23 cm^3^ (*p* = 0.28). In the study of Kendel Jovanović et al. (24), patients followed an energy-reduced anti-inflammatory diet for 6-months. Based on unadjusted linear regression analysis, change in VAT was not significantly associated with a decrease in the fatty liver index (FLI; β −0.21, *p* = 0.308) and NAFLD liver fat score (NAFLD-LFS; β −0.53, *p* = 0.324). Kim et al. (25) described the effect of longitudinal body fat changes on NAFLD regression. In the multivariate Cox proportional hazard model, the risk for regressed NAFLD comparing tertiles 2 and 3 vs. tertile 1 of change in VAT area were hazard ratio (HR) = 0.70 (CI: 0.42–1.16) and HR = 0.38 (CI: 0.20–0.73), respectively. Lee et al. (27) examined the effect of lifestyle interventions in living liver donors with NAFLD on the improvement of different body composition parameters and changes in steatosis. The relative reduction of the visceral fat area was the only significant independent factor associated with resolved NAFLD (odds ratio—OR = 1.03, CI: 1.01–1.05). Rachakonda et al. (29) reported a twofold higher VAT loss in patients with NAFLD resolution compared to those without [−57.23 cm^2^ (−88.63 to −25.84) vs. −26.92 cm^2^ (−52.14 to −26.92), *p* = 0.034], despite a similar degree of total body weight loss.

The details of the studies included in Figure 2 are summarized in the Supplementary Results.

### 3.4. Higher subcutaneous fat area decrease is moderately correlated with NAFLD regression

Based on two (23, 27) studies with 163 patients, the correlation between SAT change and liver steatosis change was *r* = 0.42 (0.29–0.54, Pearson's correlation, Figure 2). Further details are summarized in the Supplementary Results.

Kim et al. (25) reported on the median SAT change between the regressed and persistent NAFLD groups and found a significant difference [−10.01 cm^2^ (−31.73, 10.71) vs. +3.33 cm^2^ (−17.4, 18.20), *p* < 0.001]. They also examined the risk of NAFLD regression between the tertiles of SAT. Comparing tertiles 3 vs. 1 of change in SAT area, the risk was HR = 0.48 (CI: 0.27–0.84). However, after multiple adjustments, the risk was only marginally statistically significant. After lifestyle intervention, Lee et al. (27) found significant odds of NAFLD resolution in regards to the relative reduction of SAT area (OR = 1.04, CI: 1.01–1.07). Finally, Rachakonda et al. (29) investigated the abdominal and midthigh subcutaneous area and found no differences in the means between the NAFLD resolved vs. persistent groups.

### 3.5. Muscle mass increase is positively correlated with hepatic steatosis decrease

Correlation analysis results are included in Figure 2. Because of the heterogeneous data, we did not calculate pooled results.

Based on Kim et al. (11), the highest vs. lowest tertile of increase in skeletal muscle index (SMI) over 1-year resulted in an increased adjusted HR of 4.17 (CI = 1.90–6.17) of baseline NAFLD resolution. Furthermore, a one percent increase in SMI resulted in an adjusted HR of 1.99 (CI = 1.53–2. 59). Nachit et al. (28) found that NASH improvement (≥2 pt NASH score reduction) is associated with a decreased muscle mass (18.7 vs. 4.2%, *p* = 0.046) but not muscle density change (= Psoas density, 5.1 vs. 2%, *p* = 0.549). This means that the decrease in mass mainly was muscle fat. The group with a muscle density reduction of ≥11% had a higher rate of NASH resolution (71 vs. 33%, *p* = 0.033). They concluded that a decreased muscle fat content was associated with liver histological improvement. Osaka et al. (10) evaluated the fat-to-muscle ratio change regarding liver stiffness measurement (LSM) and CAP values. They reported a significant difference in the change in the fat-to-muscle ratio between the groups with and without LSM normalization (*p* < 0.001). Furthermore, based on regression analysis, the change in fat-to-muscle ratio was associated with the rate of change in CAP (β = 0.38, *p* < 0.001) and LSM (β = 0.21, *p* = 0.026). On the other hand, Rachakonda et al. (29) did not find a significant change in the fat-free mass (*p* = 0.131) and midthigh muscle area (*p* = 0.125) between the NAFLD resolved vs. persisted groups. Lastly, Shida et al. (30) examined the longitudinal changes in skeletal muscle mass to visceral fat area ratio (SV ratio). The increase in the CAP was significantly higher in the worsened SV ratio group (decreased by >5%, 27.9 ± 8, *p* < 0.01 compared to improved). In comparison, the improved SV ratio group was associated with a CAP decrease (increased by >5%, −20 ± 12.3, *p* < 0.01 compared to stable).

Further details are summarized in the Supplementary Results.

### 3.6. Risk of bias analysis

Overall, most studies presented a low or moderate risk of bias for the assessed domains. The “Study confounding” domain represented the lowest quality, while the study participants represented a low risk of bias (Supplementary Figure 1 and Supplementary Table 4).

## 4. Discussion

In our present systematic review, we aimed to highlight the following key observations: changes in both VAT and SAT showed a significant, moderately positive correlation with changes in fatty liver. Furthermore, increasing skeletal muscle mass and decreasing myosteatosis can also be associated with the decrease in liver steatosis. Based on the included studies, none of the body composition improvement was associated with the worsening of liver steatosis. Based on these results, investigating the interplay between VAT and SMM, it seems that the clinical course of fatty liver worsened with the increase of visceral fat coupled with a decrease in muscle mass.

Based on current guidelines, an extensive lifestyle adjustment is mandatory and remains the cornerstone management of NAFLD. All practice guidelines emphasize energy restriction and physical activity, promoting weight loss as the keynote endpoint. Although a consensus is reached that all interventions should be gradual and individually tailored, no recommendation exists for using body composition analysis (3). On the other hand, guidelines do not yet recommend the body composition analysis at baseline (2).

Patients with obesity have a high risk of NAFLD, and a central fat distribution, in particular, is a strong independent predictor of mortality. The lean-NAFLD population further confirms this, in which a normal BMI meets the nocuous metabolic pattern associated with increased VAT and insulin resistance (32). Several differences between VAT and SAT explain why VAT is associated with more metabolically adverse features than SAT (33). Visceral obesity seems to be a metabolically active endocrine organ and is responsible for the overflow of free fatty acids into the bloodstream, promoting its accumulation in ectopic sites, such as the liver. SAT seems to be more of an inert reservoir of fat (8). We also have to consider gender differences, recognizing that men are more prone to VAT than SAT, whereas women are the other way around due to their metabolic and hormone profiles (34). It should be noted that the NAFLD resolution was rather achieved with a significant VAT decrease, despite an identical extent of weight or total fat loss (24). With all this in mind, reducing the amount of these fat types means not only decreased weight (which in itself has benefits) but leads to major metabolic improvements that can promote further decrease in steatosis- and histological regression (35).

Based on Kendel Jovanović et al. (24) the group with a significant VAT reduction achieved impressive improvements in liver steatosis and fibrosis compared to the group with a reduction of the total fat mass. Although, the average weight loss was the same for both groups. Rachakonda et al. (29) explored the impact of altering fat-free mass and fat mass and found that subjects with greater declines in fat mass achieved higher rates of fatty liver regression associated with notable VAT loss.

Another influential body composition parameter is skeletal muscle mass. Its importance is increasingly recognized, especially in adipose-muscle-liver axis dysfunction. Abnormal endocrine signaling back and forth between expanded fat depots and fatty infiltrated liver and muscle leads to insulin resistance. This hyperinsulinemic state continuously worsens steatosis (36). The number of longitudinal studies investigating potential correlations between NAFLD improvement and SMM increase is scarce, but as far as this review results, improved muscle mass and function are likely to benefit NAFLD outcomes. This impact is dose-dependent and promotes whole health with combined advantages. Specific interventions targeting SMM increase also contribute to improving SMM density, likely to have a metabolically therapeutic effect (28).

Kim et al. (11) studied the individual influence of skeletal muscle on fatty liver change and found that the cumulative incidence of NAFLD resolution was significantly higher in patients in the highest tertile of change over 1 year, compared with the lowest tertiles, even after adjustment of covariates. On the other hand, Shida et al. (30) and Jiang et al. (37) focused on the interplay between visceral fat and skeletal muscle mass, and both studied the association between altering the SV ratio (skeletal muscle to visceral fat). The shared conclusion is that this combined index could be favored because it simultaneously describes variations in both parameters. The clinical course of fatty liver worsened with a decreased SV ratio, which means increased visceral fat and decreased muscle mass.

Currently, lifestyle modifications are at hand to influence different body composition parameters. However, they should be designed on an individual basis. Weight loss induced only by caloric restriction results in fat tissue and fat-free mass loss (38). Physical activity should be simultaneously sustained for absolute or, in some instances, relative SMM increase. The optimal approach of the former or the latter should be as customized as possible and adjusted to be sustainable on the long run. On the other hand, these results may help to identify future therapeutic targets. For example, the results of the currently invesitgated glucagon-like peptide-1 (GLP-1) with glucose-dependent insulinotropic polypeptide agonism (tirzepatide) or GLP-1 with glucagon agonism (cotadutide) may be enhanced with proper lifestyle modifications (39).

### 4.1. Strengths and limitations

The strength of our study is the rigorous methodology we used, although we supplemented the inclusion criteria of the studies. In addition, the included studies originated from multiple countries and were prospective cohort studies. On the other hand, the most important limitation of our study is the low number of quantitative analyses. Consequently, there was significant heterogeneity in the study population, body composition, and liver steatosis measurements. However, we managed to include a wide range of results. Most included studies consisted of small cohorts of patients with various follow-up intervals. In addition, we included studies with multiple lifestyle interventions.

## 5. Conclusion

Based on the current literature, besides weight loss, the maintenance of functionally healthy muscle mass and a decrease in VAT and SAT may be associated with a decrease in liver steatosis. However, more homogenous results are needed.

## 6. Implications for practice and research

The benefit of immediate implementation of the scientific results has been already proven (40, 41).

Body composition parameters should be included in the assessment of NAFLD patients, while a medical team should manage these patients by incorporating individualized diet and exercise therapies. On the other hand, lifestyle changes should last more than a couple of months.

Randomized controlled trials are needed focusing on the body composition of NAFLD patients and investigating the effect of different diets and physical activity on different body composition parameters and NAFLD resolution.

## Data availability statement

The original contributions presented in the study are included in the article/Supplementary material, further inquiries can be directed to the corresponding author.

## Author contributions

DM: conceptualization and writing—original draft. PH: conceptualization, funding acquisition, and writing—review and editing. BT: conceptualization, visualization, and writing—original draft. TT and BE: conceptualization and writing—review and editing. GP: conceptualization, supervision, and writing—original draft. SV: conceptualization, methodology, project administration, formal analysis, and writing—original draft. All authors certify that they have participated sufficiently to take public responsibility for the content, including participation in the manuscript's concept, design, analysis, writing, or revision.
