# Supplementary material for: Improved body composition decreases the fat content in non-alcoholic fatty liver disease, a meta-analysis and systematic review of longitudinal studies
Source: Front Med (Lausanne). 2023 May 4;10:1114836. doi: 10.3389/fmed.2023.1114836 (PMC10194653; doi:10.3389/fmed.2023.1114836)
Supplement: Supplementary file 1 [file Data_Sheet_1.docx]

**SUPPLEMENTARY MATERIALS**

**TITLE**

Improved body composition decreases the fat content in non-alcoholic fatty liver disease, a meta-analysis and systematic review of longitudinal studies

**AUTHORS**

Dóra Mátis^1^, Péter Hegyi^1,2,3^, Brigitta Teutsch^1,2^, Tamás Tornai^3^, Bálint Erőss^1,2,3^, Gabriella Pár^4†^, Szilárd Váncsa^1,2,3†^

**AFFILIATION**

1. Centre for Translational Medicine, Semmelweis University, Budapest, Hungary
2. Institute for Translational Medicine, Medical School, University of Pécs, Pécs, Hungary
3. Institute of Pancreatic Diseases, Semmelweis University, Budapest, Hungary
4. Division of Gastroenterology, First Department of Medicine, Medical School, University of Pécs, Pécs, Hungary;

† authors contributed equally

**TABLE OF CONTENT**

**Supplementary Table 1.** PRISMA 2020 checklist

**Supplementary Table 2.** Eligibility criteria in each included article in the systematic review and meta-analysis

**Supplementary Table 3.** The detailed description of performed interventions

**Supplementary Table 4.** Risk of bias assessment using the QUIPS risk of bias assessment tool

**Supplementary Appendix 1.** QUIPS risk of bias assessment

**Supplementary Results 1**

**Supplementary Figure 1.** QUIPS risk of bias assessment summary plot

**Supplementary Table 1.** PRISMA 2020 checklist (1)

| **Section and topic** | **Item #** | **Checklist item** | **Location where item is reported** |
| --- | --- | --- | --- |
| **Title** | | | |
| Title | 1 | Identify the report as a systematic review. | 1 |
| **Abstract** | | | |
| Abstract | 2 | See the PRISMA 2020 for Abstracts checklist (table 2). | 1 |
| **Introduction** | | | |
| Rationale | 3 | Describe the rationale for the review in the context of existing knowledge. | 2 |
| Objectives | 4 | Provide an explicit statement of the objective(s) or question(s) the review addresses. | 2 |
| **Methods** | | | |
| Eligibility criteria | 5 | Specify the inclusion and exclusion criteria for the review and how studies were grouped for the syntheses. | 3 |
| Information sources | 6 | Specify all databases, registers, websites, organisations, reference lists and other sources searched or consulted to identify studies. Specify the date when each source was last searched or consulted. | 3 |
| Search strategy | 7 | Present the full search strategies for all databases, registers and websites, including any filters and limits used. | 3 |
| Selection process | 8 | Specify the methods used to decide whether a study met the inclusion criteria of the review, including how many reviewers screened each record and each report retrieved, whether they worked independently, and if applicable, details of automation tools used in the process. | 3 |
| Data collection process | 9 | Specify the methods used to collect data from reports, including how many reviewers collected data from each report, whether they worked independently, any processes for obtaining or confirming data from study investigators, and if applicable, details of automation tools used in the process. | 4 |
| Data items | 10a | List and define all outcomes for which data were sought. Specify whether all results that were compatible with each outcome domain in each study were sought (e.g. for all measures, time points, analyses), and if not, the methods used to decide which results to collect. | 4 |
|  | 10b | List and define all other variables for which data were sought (e.g. participant and intervention characteristics, funding sources). Describe any assumptions made about any missing or unclear information. | 4 |
| Study risk of bias assessment | 11 | Specify the methods used to assess risk of bias in the included studies, including details of the tool(s) used, how many reviewers assessed each study and whether they worked independently, and if applicable, details of automation tools used in the process. | 4 |
| Effect measures | 12 | Specify for each outcome the effect measure(s) (e.g. risk ratio, mean difference) used in the synthesis or presentation of results. | 4 |
| Synthesis methods | 13a | Describe the processes used to decide which studies were eligible for each synthesis (e.g. tabulating the study intervention characteristics and comparing against the planned groups for each synthesis (item #5)). | 4 |
|  | 13b | Describe any methods required to prepare the data for presentation or synthesis, such as handling of missing summary statistics, or data conversions. | 4 |
|  | 13c | Describe any methods used to tabulate or visually display results of individual studies and syntheses. | 4 |
|  | 13d | Describe any methods used to synthesise results and provide a rationale for the choice(s). If meta-analysis was performed, describe the model(s), method(s) to identify the presence and extent of statistical heterogeneity, and software package(s) used. | 4 |
|  | 13e | Describe any methods used to explore possible causes of heterogeneity among study results (e.g. subgroup analysis, meta-regression). | 4 |
|  | 13f | Describe any sensitivity analyses conducted to assess robustness of the synthesised results. | 4 |
| Reporting bias assessment | 14 | Describe any methods used to assess risk of bias due to missing results in a synthesis (arising from reporting biases). | 4 |
| Certainty assessment | 15 | Describe any methods used to assess certainty (or confidence) in the body of evidence for an outcome. | - |
| **Results** | | | |
| Study selection | 16a | Describe the results of the search and selection process, from the number of records identified in the search to the number of studies included in the review, ideally using a flow diagram (see fig 1). | 4 |
|  | 16b | Cite studies that might appear to meet the inclusion criteria, but which were excluded, and explain why they were excluded. | 4 |
| Study characteristics | 17 | Cite each included study and present its characteristics. | 4, Table 1 |
| Risk of bias in studies | 18 | Present assessments of risk of bias for each included study. | 6 |
| Results of individual studies | 19 | For all outcomes, present, for each study: (a) summary statistics for each group (where appropriate) and (b) an effect estimate and its precision (e.g. confidence/credible interval), ideally using structured tables or plots. | 5-6, Supplement |
| Results of syntheses | 20a | For each synthesis, briefly summarise the characteristics and risk of bias among contributing studies. | 5-6, Supplement |
|  | 20b | Present results of all statistical syntheses conducted. If meta-analysis was done, present for each the summary estimate and its precision (e.g. confidence/credible interval) and measures of statistical heterogeneity. If comparing groups, describe the direction of the effect. | 5-6, Supplement |
|  | 20c | Present results of all investigations of possible causes of heterogeneity among study results. | 5-6, Supplement |
|  | 20d | Present results of all sensitivity analyses conducted to assess the robustness of the synthesised results. | 5-6, Supplement |
| Reporting biases | 21 | Present assessments of risk of bias due to missing results (arising from reporting biases) for each synthesis assessed. | - |
| Certainty of evidence | 22 | Present assessments of certainty (or confidence) in the body of evidence for each outcome assessed. | - |
| **Discussion** | | | |
| Discussion | 23a | Provide a general interpretation of the results in the context of other evidence. | 6-7 |
|  | 23b | Discuss any limitations of the evidence included in the review. | 8 |
|  | 23c | Discuss any limitations of the review processes used. | 8 |
|  | 23d | Discuss implications of the results for practice, policy, and future research. | 8 |
| **Other information** | | | |
| Registration and protocol | 24a | Provide registration information for the review, including register name and registration number, or state that the review was not registered. | CRD42021278584 |
|  | 24b | Indicate where the review protocol can be accessed, or state that a protocol was not prepared. | PROSPERO |
|  | 24c | Describe and explain any amendments to information provided at registration or in the protocol. | Methods |
| Support | 25 | Describe sources of financial or non-financial support for the review, and the role of the funders or sponsors in the review. | 8 |
| Competing interests | 26 | Declare any competing interests of review authors. | 8 |
| Availability of data, code, and other materials | 27 | Report which of the following are publicly available and where they can be found: template data collection forms; data extracted from included studies; data used for all analyses; analytic code; any other materials used in the review. | 9 |

**Supplementary Table 2.** Eligibility criteria in each included article in the systematic review and meta-analysis

| **Study** | **Inclusion criteria** | **Exclusion criteria** |
| --- | --- | --- |
| Charatcharoenwitthaya et al. (2021)(2) | “All study participants were medical personnel who were recruited using flyers and posters placed on campus. Sedentary individuals with the absence of significant alcohol consumption (alcohol intake ,20 g/d for women or ,30 g/d for men) were invited to attend an ultrasonographic examination. Subjects who had bright liver on ultra-sonography were asked to confirm a diagnosis of liver steatosis with transient elastography using the controlled attenuation parameter (CAP) .248 dB/m (7).” | “Subjects were excluded if they had evidence of viral hepatitis, autoimmune liver disease, hemochromatosis, or drug-induced hepatotoxicity; they were engaging in weight loss program or structured physical activity for the previous 3 months; they had medical conditions preventing participation in the exercise program; or used drugs known to influence glucose metabolism and body composition.” |
| Cuthbertson et al. (2016)(3) | “Inclusion criteria were a diagnosis of NAFLD, being sedentary (<2 h/week low-intensity physical activity, no moderate- or high-intensity activity), non-smokers, with alcohol consumption < 14 (females) and < 21 (males) units/week.” | “Exclusion criteria were T2DM, ischaemic heart disease or contraindications to exercise. Participants were excluded from follow-up assessment if they deviated from their habitual diet and lost excessive weight.” |
| Houghton et al. (2017)(4) | “Thirty-one patients with histologically characterised NASH (age 59 ± 12years, BMI 35 ±5kg/m^2^) were screened for study entry.” | “Patients with evidence of other liver disease or a history of excessive alcohol consumption (alcohol intake >20g/day for women; >30g/day for men) were excluded. Other exclusion criteria included: heart or kidney disease; implanted ferrous metal; pre-existing medical conditions preventing participation in the exercise programme; insulin sensitizing treatment or dietary change over the preceeding six months.” |
| Huang et al. (2005)(5) | “Adult patients with a liver biopsy demonstrating NASH within 1 year of enrollment, a BMI greater than 25 kg/m2, no other etiologies of chronic liver disease, alcohol use <20 g a day, and an elevation in serum aspartate aminotransferase (AST) or alanine aminotransferase (ALT) above the upper limit of normal on at least one occasion as defined by our hospital laboratory manual were included. “ | “Patients with other causes of chronic liver disease including viral hepatitis, autoimmune hepatitis, cholestatic liver disease, alpha-1 antitrypsin deficiency, hemochromatosis, Wilson’s disease, and alcoholic liver disease were excluded based on history, laboratory results, and histologic features. The patients on medications known to cause hepatic steatosis, had undergone weight reduction surgery within the past year, or used weight loss medications within 3 months of evaluation, were also excluded from participation in this study.” |
| Jovanovic et al. (2021)(6) | “The inclusion criteria were an age of 18 to 50 years, BMI ≥ 30 kg/m2 with or without obesity-related complications, and stable body weight for the previous three months.” | “Exclusion criteria were cigarette smoking within 6 months before study initiation, chronic heart, kidney, and/or severe liver disease, malignant disease or history of malignant disease, use of anti-inflammatory or immunosuppressive drugs or medications for weight loss, changes in chronic medications, active infection or surgicalprocedure in the previous three months, food allergy or intolerance to any anti-inflammatory diet constituent, pregnancy, and lactation.” |
| Keating et al. (2015)(7) | “Inactive (exercising <3 days/week) and overweight or obese (BMI >25 kg/m2) adult (29 to 59 year-old) men and women were randomized into one of four arms involving either 8 weeks of: HI:LO, LO:HI, LO:LO, or PLA exercise intervention from August 2011 to October 2013.” | “Volunteers were excluded if taking lipid-lowering or insulin sensitizing medication, reported a high alcohol intake (>20 g/day), had secondary causes of steatohepatitis, alcoholic liver disease or viral hepatitis.” |
| Kim D et al. (2017)(8) | “Briefly, between March 2007 and December 2008, a total of 3718 subjects without liver disease and/or significant alcohol consumption (>30 g/day for men and >20 g/day for women) [1] were initially included in the Gangnam NAFLD cohort.” | “We excluded 1701 subjects who did not attend any voluntary follow-up health screenings between 2011 and 2013.” |
| Kim G et al. (2018)(9) | “We enrolled 125 patients who visited Tottori University Hospital between January 1996 and October 2005. A questionnaire about their personal medical history, including alcohol intake, was given. The history of alcohol intake was determined as follows: never drink, occasionally drink (1–4 times per week), or drink almost every day (at least 23g/day of alcohol intake 5–7 times per week).” | “We excluded patients who drank almost every day, were hepatitis C virus antibody positive and hepatitis B surface antigen positive, and had a medical history of autoimmune epatitis, primary biliary cirrhosis, or primary sclerosing cholangitis.” |
| Koda et al. (2007)(10) | “We enrolled 20,069 subjects age 20 years or older who underwent four or more follow-up comprehensive health examinations either annually or biennially from August 2006 through August 2013 at the Health Promotion Center at Samsung Medical Center, Seoul, Republic of Korea. The study population consisted of employees of various organizations and companies, and subjects who voluntarily took part in the annual or biennial comprehensive health screening examinations at the center.” | “Subjects with missing data for baseline skeletal muscle mass (n = 355), waist circumference, body weight, or laboratory results (n = 1294), and subjects with positive serologic markers of hepatitis B virus (n = 822) or hepatitis C (n = 161) or daily alcohol consumption greater than 30 g for men and greater than 20 g for women (n = 1039) or missing data for alcohol consumption (n = 851) were excluded. “ |
| Lee et al. (2021)(11) | “The inclusion criteria for this study were as follows: (1) initial liver biopsy findings of more than 5% HS without steatohepatitis, (2) living donor candidates who had undergone lifestyle intervention to improve HS before liver donation, and (3) availability of follow-up abdominal CT imaging and liver biopsy results after lifestyle intervention and before liver donation.” | “Subjects for whom the time interval between paired CT examinations and liver biopsies was > 3 months (n = 41) were excluded (Fig. 1).” |
| Nachit et al. (2021)(12) | “Briefly, overweight or obese patients visiting the obesity clinic underwent a metabolic and a liver-specific work-up including a detailed questionnaire, a bio-impedance analysis (BIA) and a CT scan at L4 level.” | “Patients were excluded in case of a liver disease other than NAFLD, significant alcohol consumption (>20 g/day), a history of bariatric surgery or pre-existing diabetes. Since diabetes is a specific risk factor for NASH and fibrosis and some drugs used to treat diabetes may beneficially impact NAFLD histology, introducing potential substantial confounding,25 pre-existing diabetes (defined as an established diagnosis on previous assessment and/or active use of antidiabetic drugs) was an exclusion criterion.” |
| Osaka et al. (2019)(13) | “…type 2 diabetes patients with NAFLD. We included the patients who underwent both elastography and bioelectrical impedance body composition evaluations.” | “We excluded the patients without NAFLD, the definition is described below. Exclusion criteria was set as no data of lever stiness measurement, or no data of bio impedance analysis.” |
| Rachakonda et al. (2017)(14) | “Inclusion criteria included World Health Organization Class II or III obesity (defined as BMI 35 kg/m2), ability to walk without assistance, and ability to obtain medical clearance for dietary and physical activity interventions.” | “Exclusion criteria included history of coronary artery disease, diagnosis of cancer within 5 years of enrollment, prior bariatric surgery, prior participation in a weight loss program within 1 year of enrollment, history of diabetes mellitus, uncontrolled hypertension, and pregnancy within 6 months of enrollment. Participants with liver enzyme elevations more than 30% above the upper limit of normal laboratory ranges were excluded.” |
| Shida et al. (2019)(15) | “... who were referred to the Tsukuba  University Hospital outpatient department for the first time from April 2011 to October 2015 after hepatic dysfunction or fatty liver was detected as a result of a complete medical checkup or medical examination, were diagnosed with NAFLD.” | “The patients were removed from this study as follows: (a) the presence of other causes of liver disease, (b) psychiatric disease, and (c) those who were taking anti-diabetic or weight loss medication. Patients with an alcohol consumption of >20 g/day were also excluded.” |
| Takahashi et al. (2017)(16) | “The diagnosis of NAFLD was based on the Asia-Pacific Working Party guidelines for NAFLD.” | “Exclusion criteria comprised: evidence of other liver diseases such as chronic hepatitis C, chronic hepatitis B, autoimmune hepatitis, primary biliary cirrhosis or alcoholic liver disease ( > 20 g of alcohol/day);consumption of weight-loss agents, corticosteroids, tamoxifen, herbal medicines or anti-diabetic agents (patients with metabolic complications who were stable for > 6 months without additional drugs included); or heart or kidney disease or other pre-existing medical conditions that might prevent participation in the exercise program.” |

**Supplementary Table 3.** The detailed descriptions of performed interventions

| **Author (year)** | **Intervention** |
| --- | --- |
| Charatcharoenwitthaya et al. (2021)(2) | “Eighteen subjects exercised for an average of 3.35 ± 0.30 sessions a week in the aerobic group, and 17 subjects exercised an average of 3.39 ± 0.28 sessions a week in the resistance group.” |
| Cuthbertson et al. (2016)(3) | “NAFLD patients were randomized to 16 weeks of exercise supervision (n =38) or counseling (n = 31) without dietary modification.” |
| Houghton et al. (2017)(4) | “Twenty-four patients (mean age, 52 ± 14 y; body mass index, 33 ± 6 kg/m2) with sedentary lifestyles (<60 min/wk of moderate-vigorous activity) and biopsy-proven NASH were assigned randomly to groups that exercised (n = 12) or continued standard care (controls, n=12) for 12 weeks while maintaining their weight.” |
| Huang et al. (2005)(5) | “All participants received standardized nutritional counseling directed at decreasing IR and promoting gradual weight loss (1–2 pounds/wk) over the duration of the study.” |
| Jovanovic et al. (2021)(6) | “..participants were randomly assigned to the anti-inflammatory diet (AID) group or the control diet (CD) group. - Each dietary intervention has been described in a study protocol. “ |
| Keating et al. (2015)(7) | “Inactive and overweight/obese adults received 8 weeks of either; i) low to moderate intensity, high volume aerobic exercise (LO:HI, 50% VO2peak, 60 min, 4 d/week); ii) high intensity, low volume aerobic exercise (HI:LO, 70% VO2peak, 45 min, 3 d/week); iii) low to moderate intensity, low volume aerobic exercise (LO:LO, 50% VO2peak, 45 min, 3 d/week); or iv) placebo (PLA).” |
| Kim D et al. (2017)(8) | None |
| Kim G et al. (2018)(9) | None |
| Koda et al. (2007)(10) | None |
| Lee et al. (2021)(11) | “The subjects underwent a dietary control and exercise regimen.” |
| Nachit et al. (2021)(12) | “All had a therapeutic intervention consisting of dietary counseling or bariatric surgery.” |
| Osaka et al. (2019)(13) | None |
| Rachakonda et al. (2017)(14) | “One-year intensive lifestyle intervention consisting of diet and physical activity. One group (initial physical activity) was randomized to diet and physical activity for the entire 12 months; the other group (delayed physical activity) had the identical dietary intervention but with physical activity delayed for 6 months.” |
| Shida et al. (2019)(15) | None |
| Takahashi et al. (2017)(16) | “Patients in the exercise group were educated about resistance exercises comprising push-ups and squats at the beginning of the study. They then performed 3 sets of 10 push-ups and 3 sets of 10 squats with a 1-min interval between each set over a period of 20–30min, and recorded their compliance with the regimen.” |

**Supplementary Table 4.** Risk of bias assessment using the QUIPS risk of bias assessment tool(17)

| **Author (year)** | **1** | **2** | **3** | **4** | **5** | **6** |
| --- | --- | --- | --- | --- | --- | --- |
| Charatcharoenwitthaya et al. (2021)(2) |  | N/A |  |  |  |  |
| Cuthbertson et al. (2016)(3) |  | N/A |  |  |  |  |
| Houghton et al. (2017)(4) |  |  |  |  |  |  |
| Huang et al. (2005)(5) |  |  |  |  |  |  |
| Jovanovic et al. (2021)(6) |  | N/A |  |  |  |  |
| Keating et al. (2015)(7) |  |  |  |  |  |  |
| Kim D et al. (2017)(8) |  | N/A |  |  |  |  |
| Kim G et al. (2018)(9) |  | N/A |  |  |  |  |
| Koda et al. (2007)(10) |  | N/A |  |  |  |  |
| Lee et al. (2021)(11) |  | N/A |  |  |  |  |
| Nachit et al. (2021)(12) |  |  |  |  |  |  |
| Osaka et al. (2019)(13) |  | N/A |  |  |  |  |
| Rachakonda et al. (2017)(14) |  | N/A |  |  |  |  |
| Shida et al. (2019)(15) |  | N/A |  |  |  |  |
| Takahashi et al. (2017)(16) |  | N/A |  |  |  |  |

**N/A:** not attributable

**Items in columns** **1:** Study participation, **2:** Study attrition, **3:** Prognostic factor measurement, **4:** Outcome measurement, **5:** Study confounding, **6:** Statistical analysis and reporting

**Colors represent: Green:** low risk of bias, **Yellow:** moderate risk of bias, **Red:** high risk of bias

**Supplementary Appendix 1.** QUIPS risk of bias assessment (17)

Overall ratings for each domain were assigned as carrying ‘low’ (green), ‘moderate’ (yellow), or ‘high’ (red) risk of bias, based on the items included in each domain.

**Study participation measurement:** (1) low risk of bias was attributed if authors adequately described the source population, including methods to identify patients and eligibility criteria. The description of the time and place of recruitment were also added to low risk of bias; (2) moderate risk of bias was attributed if a part of the above-listed descriptions were missing; (3) high risk of bias was attributed if baseline characteristics, eligibility criteria, time and place of recruitment were not described.

**Study attrition assessment** was performed in the case of prospective studies: (1) low risk of bias was attributed if the proportion of baseline sample was available, also if the reason for lost to follow-up was detailed; (2) moderate risk of bias was attributed if a part of the above-listed criteria were missing; (3) high risk of bias was attributed if data was missing for the above-mentioned criteria. We did not assess attrition bias in retrospective studies (N/A – not attributable).

**Prognostic factor measurement:** (1) low risk of bias was attributed if the definition of the body composition was based on clear criteria; (2) moderate risk of bias was attributed if the diagnosis of body composition was acquired from anamnesis; (3) high risk of bias was attributed if there was no definition provided for body composition.

**Outcome measurement:** (1) low risk of bias was attributed if the patients were followed up regularly after enrollment; (2) moderate risk of bias was attributed if a part of the above-mentioned criteria were missing; (3) high risk of bias was attributed if the follow-up policy was not described or it was inadequate.

**Study confounding measurement:** (1) low risk of bias was attributed if important potential confounders were described and accounted for in the analysis (i.e., appropriate adjustment); (2) moderate risk of bias was attributed if some of the important confounders were not measured; (3) high risk of bias was attributed if studies did not provide data on confounding factors.

**Statistical analysis measurement** (1) low risk of bias was attributed if the used methodology was clearly described; (2) moderate risk of bias was attributed if the statistical analysis was not clearly described; (3) high risk of bias was attributed if the used methodology was not described.

**Supplementary Results**

**Visceral adipose tissue area decrease correlates with steatosis improvement**

Charatcharoenwitthaya et al.(2) examined the effect of aerobic and resistance exercise. Based on the combined data, reduction in hepatic fat content after training was positively associated with changes in visceral fat rating (r=0.61, *p*<0.001). Cuthbertson et al.(3) investigated obese patients with NAFLD. After 16 weeks of exercise supervision or counseling, they reported an average correlation between intrahepatocellular lipid (IHCL) and VAT volume reduction (r=0.37, *p*=0.008). Houghton et al.(4) recruited patients with NASH for a 12-week aerobic and resistance training. They reported a positive correlation between the changes in hepatic triglyceride content (HTGC) and VAT after the intervention (12 patients, r=0.39, *p*=0.03). Keating et al.(7) examined the effect of different aerobic exercise training doses on liver fat and visceral adiposity. Correlations were performed using combined data from all study participants. Change in IHL was significantly correlated with VAT (r=0.435, *p*=0.002). Koda et al.(10) reported on the longitudinal assessment of body fat distribution and hepatic steatosis. Correlation analysis was carried out by univariate linear regression analysis. The VAT thickness change significantly correlated with the hepatic steatosis score change (r=0.741, *p*<0.001). Lee et al.(11) presented the results of VAT and SAT measured on CT examinations before and after lifestyle intervention in living liver donors with NAFL. Results showed a weak correlation between the relative changes in hepatic steatosis and VAT (r=0.278, *p*=0.003).

**Higher subcutaneous fat area decrease is associated with NAFLD regression**

Based on Cuthbertson et al.(3), there was a strong correlation between reduction in IHCL and reduction in SAT volume (r=0.61, *p*<0.001). In the study by Keating et al.(7), correlations were performed using combined data from all study participants. Change in IHL was significantly correlated with SAT (r=0.517, *p*<0.001). Furthermore, Koda et al.(10) reported a significant positive correlation between change in SAT and hepatic steatosis score improvement (r=0.741, *p*<0.001).Lastly, Lee et al.(11) reported a weak correlation between the relative changes in HS by liver biopsy and the relative changes in SAT area measured on CT examinations (r=0.382, *p*<0.001).

**Body fat area decrease is positively associated with a reduction in hepatic fat**

Based on Charatcharoenwitthaya et al.(2), reduction in hepatic fat content after training was positively associated with changes in body fat mass (r=0.52, *p*=0.001). Huang et al.(5) reported on the mean change in body fat by body impedance analysis and found a non-significant difference between groups with and without NASH score (0-17) improvement (0.63 vs. 1.1, *p*=0.82). In the study of Jovanovic et al.(6), patients followed an energy-reduced anti-inflammatory diet for 6-months. Based on unadjusted linear regression analysis, change in VAT was not significantly associated with a decrease in the fatty liver index (FLI) (β -0.09, *p*=0.265), NAFLD liver fat score (NAFLD-LFS) (β -0.21, *p*=0.282), and Fib4 score (β -0.35, *p*=0.07). Finally, based on Rachakonda et al.(14), fat mass change in kg was non significantly different between the NAFLD resolved vs. persist groups [-10.58 kg (-13.6 to -7.57) vs. -7.23 kg (-9.61 to -4.84), *p*=0.489].

**Muscle mass increase is positively correlated with hepatic steatosis decrease**

Charatcharoenwitthaya et al.(2) reported a moderate correlation between change in total muscle mass and hepatic fat content: r=0.42; *p*=0.012. On the other hand, Lee et al.(11) observed a very weak positive correlation between skeletal muscle area percentage change and hepatic steatosis change (r=0.152, *p*=0.11). In the same study, the odds of NAFLD resolution were higher regarding the relative reduction of SMA (OR=1.12, CI: 1.012–1.227). Takahashi et al.(16) investigated the association between change in muscle/body weight ratio and absolute change in alanine aminotransferase (ALT) after 24 weeks of resistance exercise. Based on their results, a lower muscle/ body weight ratio resulted in ALT increase (r=-0.374, *p*=0.049).


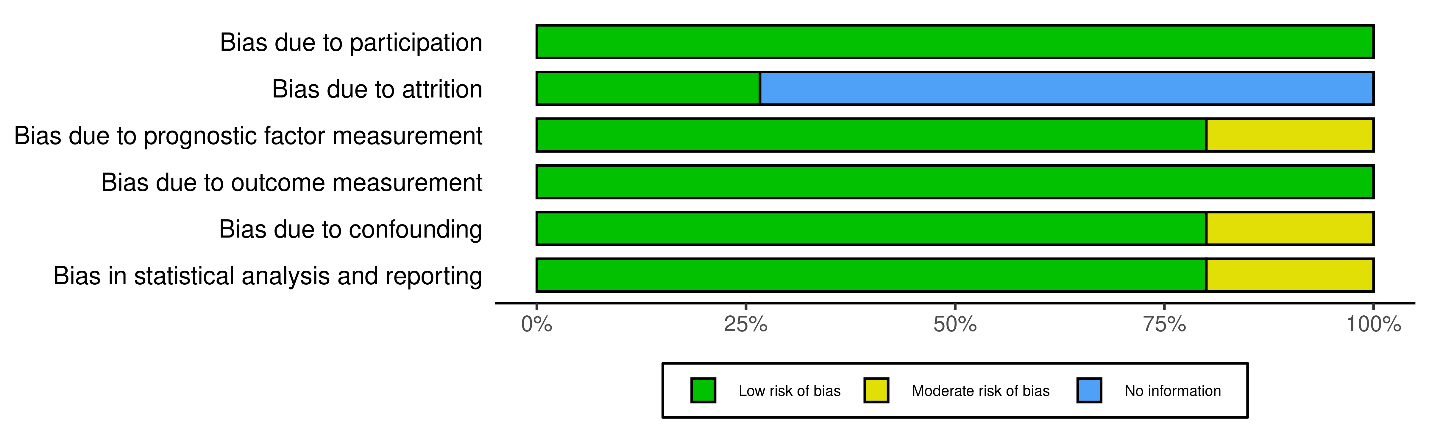
**Supplementary Figure 1.** QUIPS risk of bias assessment summary plot

**REFERENCE**

1. Page MJ, McKenzie JE, Bossuyt PM, Boutron I, Hoffmann TC, Mulrow CD, et al. The PRISMA 2020 statement: an updated guideline for reporting systematic reviews. *BMJ*. (2021) 372:n71. doi: 10.1136/bmj.n71

2. Charatcharoenwitthaya P, Kuljiratitikal K, Aksornchanya O, Chaiyasoot K, Bandidniyamanon W, Charatcharoenwitthaya N. Moderate-Intensity Aerobic vs Resistance Exercise and Dietary Modification in Patients With Nonalcoholic Fatty Liver Disease: A Randomized Clinical Trial. *Clinical and Translational Gastroenterology*. (2021) 12(3).

3. Cuthbertson DJ, Shojaee-Moradie F, Sprung VS, Jones H, Pugh CJ, Richardson P, et al. Dissociation between exercise-induced reduction in liver fat and changes in hepatic and peripheral glucose homoeostasis in obese patients with non-alcoholic fatty liver disease. *Clin Sci (Lond)*. (2016) 130(2):93-104. doi: 10.1042/cs20150447

4. Houghton D, Thoma C, Hallsworth K, Cassidy S, Hardy T, Burt AD, et al. Exercise Reduces Liver Lipids and Visceral Adiposity in Patients With Nonalcoholic Steatohepatitis in a Randomized Controlled Trial. *Clin Gastroenterol Hepatol*. (2017) 15(1):96-102.e3. doi: 10.1016/j.cgh.2016.07.031

5. Huang MA, Greenson JK, Chao C, Anderson L, Peterman D, Jacobson J, et al. One-year intense nutritional counseling results in histological improvement in patients with non-alcoholic steatohepatitis: a pilot study. *Am J Gastroenterol*. (2005) 100(5):1072-81. doi: 10.1111/j.1572-0241.2005.41334.x

6. Kenđel Jovanović G, Mrakovcic-Sutic I, Pavičić Žeželj S, Benjak Horvat I, Šuša L, Rahelić D, et al. Metabolic and Hepatic Effects of Energy-Reduced Anti-Inflammatory Diet in Younger Adults with Obesity. *Canadian Journal of Gastroenterology and Hepatology*. (2021) 2021:6649142. doi: 10.1155/2021/6649142

7. Keating SE, Hackett DA, Parker HM, O’Connor HT, Gerofi JA, Sainsbury A, et al. Effect of aerobic exercise training dose on liver fat and visceral adiposity. *Journal of Hepatology*. (2015) 63(1):174-82. doi: 10.1016/j.jhep.2015.02.022

8. Kim D, Chung GE, Kwak M-S, Kim YJ, Yoon J-H. Effect of longitudinal changes of body fat on the incidence and regression of nonalcoholic fatty liver disease. *Digestive and Liver Disease*. (2018) 50(4):389-95. doi: 10.1016/j.dld.2017.12.014

9. Kim G, Lee S-E, Lee Y-B, Jun JE, Ahn J, Bae JC, et al. Relationship Between Relative Skeletal Muscle Mass and Nonalcoholic Fatty Liver Disease: A 7-Year Longitudinal Study. *Hepatology*. (2018) 68(5):1755-68. doi: <https://doi.org/10.1002/hep.30049>

10. Koda M, Kawakami M, Murawaki Y, Senda M. The impact of visceral fat in nonalcoholic fatty liver disease: cross-sectional and longitudinal studies. *Journal of Gastroenterology*. (2007) 42(11):897-903. doi: 10.1007/s00535-007-2107-z

11. Lee S, Kim KW, Lee J, Park T, Park HJ, Song GW, et al. Reduction of Visceral Adiposity as a Predictor for Resolution of Nonalcoholic Fatty Liver in Potential Living Liver Donors. *Liver Transpl*. (2021) 27(10):1424-31. doi: 10.1002/lt.26071

12. Nachit M, Kwanten WJ, Thissen JP, Op De Beeck B, Van Gaal L, Vonghia L, et al. Muscle fat content is strongly associated with NASH: A longitudinal study in patients with morbid obesity. *J Hepatol*. (2021) 75(2):292-301. doi: 10.1016/j.jhep.2021.02.037

13. Osaka T, Hashimoto YO, Takuro, Fukuda T, Yamazaki MH, Masahide, Fukui M. Reduction of Fat to Muscle Mass Ratio Is Associated with Improvement of Liver Stiness in Diabetic Patients with Non-Alcoholic Fatty Liver Disease. *Journal of Clinical Medicine* [Internet]. (2019; 8(12).

14. Rachakonda V, Wills R, DeLany JP, Kershaw EE, Behari J. Differential Impact of Weight Loss on Nonalcoholic Fatty Liver Resolution in a North American Cohort with Obesity. *Obesity (Silver Spring)*. (2017) 25(8):1360-8. doi: 10.1002/oby.21890

15. Shida T, Oshida N, Oh S, Okada K, Shoda J. Progressive reduction in skeletal muscle mass to visceral fat area ratio is associated with a worsening of the hepatic conditions of non-alcoholic fatty liver disease. *Diabetes Metab Syndr Obes*. (2019) 12:495-503. doi: 10.2147/dmso.S185705

16. Takahashi A, Imaizumi H, Hayashi M, Okai K, Abe K, Usami K, et al. Simple Resistance Exercise for 24 Weeks Decreases Alanine Aminotransferase Levels in Patients with Non-Alcoholic Fatty Liver Disease. *Sports Med Int Open*. (2017) 1(1):E2-e7. doi: 10.1055/s-0042-117875

17. Hayden JA, van der Windt DA, Cartwright JL, Côté P, Bombardier C. Assessing bias in studies of prognostic factors. *Ann Intern Med*. (2013) 158(4):280-6. doi: 10.7326/0003-4819-158-4-201302190-00009
